# Functional Analyses of Bitter Taste Receptors in Domestic Cats (*Felis catus*)

**DOI:** 10.1371/journal.pone.0139670

**Published:** 2015-10-21

**Authors:** Weiwei Lei, Aurore Ravoninjohary, Xia Li, Robert F. Margolskee, Danielle R. Reed, Gary K. Beauchamp, Peihua Jiang

**Affiliations:** 1 Monell Chemical Senses Center, 3500 Market Street, Philadelphia, Pennsylvania, 19104, United States of America; 2 Cincinnati Children’s Hospital, Cincinnati, Ohio, 45229, United States of America; German Institute of Human Nutrition Potsdam-Rehbruecke, GERMANY

## Abstract

Cats are obligate carnivores and under most circumstances eat only animal products. Owing to the pseudogenization of one of two subunits of the sweet receptor gene, they are indifferent to sweeteners, presumably having no need to detect plant-based sugars in their diet. Following this reasoning and a recent report of a positive correlation between the proportion of dietary plants and the number of *Tas2r* (bitter receptor) genes in vertebrate species, we tested the hypothesis that if bitter perception exists primarily to protect animals from poisonous plant compounds, the genome of the domestic cat (*Felis catus*) should have lost functional bitter receptors and they should also have reduced bitter receptor function. To test functionality of cat bitter receptors, we expressed cat Tas2R receptors in cell-based assays. We found that they have at least 7 functional receptors with distinct receptive ranges, showing many similarities, along with some differences, with human bitter receptors. To provide a comparative perspective, we compared the cat repertoire of intact receptors with those of a restricted number of members of the order Carnivora, with a range of dietary habits as reported in the literature. The numbers of functional bitter receptors in the terrestrial Carnivora we examined, including omnivorous and herbivorous species, were roughly comparable to that of cats thereby providing no strong support for the hypothesis that a strict meat diet influences bitter receptor number or function. Maintenance of bitter receptor function in terrestrial obligate carnivores may be due to the presence of bitter compounds in vertebrate and invertebrate prey, to the necessary role these receptors play in non-oral perception, or to other unknown factors. We also found that the two aquatic Carnivora species examined had fewer intact bitter receptors. Further comparative studies of factors driving numbers and functions of bitter taste receptors will aid in understanding the forces shaping their repertoire.

## Introduction

The so-called basic tastes (e.g., sweet and bitter) have traditionally been presumed to have evolved to ensure that animals consume an appropriate source of calories while avoiding toxic compounds [1, 2]. Although most mammals avidly consume sugars, obligate carnivores such as meat-eating cats are indifferent to them, and one component of their sweet taste receptor gene repertoire (*Tas1r2*) is a pseudogene. This loss has happened independently in several obligate carnivore species in the order Carnivora [3], presumably because there is no need to detect sweet sugars from plants. It appears that all birds have lost the Tas1r2 taste receptor gene after birds and non-avian reptiles diverged but some birds (e.g., hummingbirds) consume foods that consist primarily of sugars [4]. It was recently discovered that the nectar-eating hummingbirds have repurposed their amino acid Tas1r1/Tas1r3 receptor to detect sugars [4].

Bitter taste may provide another source of comparative evidence for an intimate relationship between diet and taste sensitivity (e.g., number of compounds and range of concentrations). Compounds that taste bitter to humans (hereafter “bitter compounds”) are widely rejected throughout the animal kingdom. It is thought that rejection is based on a mutual interaction between plants that do not “want” to be eaten and animals that do not “want” to be poisoned. A similar logic may pertain to avoidance of bitter-tasting invertebrates, reptiles and amphibians although the evidence for the extent to which cats may be exposed to such bitterness in prey as compared with exposure to plant-based bitter compounds for species that consume plants is unclear. Glendinning has argued that the relative occurrence of bitter and potentially toxic foods is lower for carnivores than for omnivores and herbivores [5].

Li and Zhang [6] have recently described a relationship between diet and the number of Tas2r genes from 54 diverse vertebrate species. Based on correlational analyses, they reported that species that tend to eat more plant food have more intact bitter receptors than species that eat little or no plant food. However, the number of functional bitter receptors may not be the only factor determining the importance of bitter taste in a species diet. For example, a species could be highly responsive to bitterness if it had a few bitter receptors that were responsive to many different bitter compounds (broadly “tuned”) as has recently been reported for the chicken [7]. Nevertheless, given the vast potential array of bitter and toxic compounds, it is hard to imagine that a few receptors could detect many or all of them. And indeed, species differ greatly in bitter receptor number. There must be a selective reason for the existence of multiple bitter receptors, particularly in light of the relative paucity of taste receptors for other modalities such as sweet, umami and probably salty and sour [1, 2].

As an initial approach to the study of bitter receptor number and function in obligate carnivores, we evaluated bitter taste receptor function in a single meat-eating obligate carnivore, the domestic cat (*Felis catus*), by expressing the receptors in cell-based assays to test their function. This strategy ensures that genes that appear to be intact by computational methods are actually functional. It also allows us to test the breadth of tuning which cannot currently be predicted with *in silico* methods. We chose the domestic cat for this study since much work has been done on sweet taste receptors in cats [8, 9] and because of the inherent interest in factors influencing feeding in this companion animal. We also sought to provide a comparative perspective by examining the broader pattern of bitter receptor number in a single order, Carnivora, in light of the question concerning the functional significance of bitter receptors in obligate carnivores.

## Results

### Domestic cats have at least seven functional bitter taste receptors

The results of nucleotide sequence searches of the feline genome indicate a total of 12 intact Tas2r receptor genes. Using cat Tas2r-specific primers, we amplified the entire coding regions of 10 of the 12 cat Tas2r receptors and inserted these receptor sequences into an expression cloning vector pCDNA3.1 (**S1 Table**). Two of the 12 receptors could not be amplified from feline DNA using primer sets designed to amplify the entire coding regions (*Tas2r1* and *Tas2r43*). We expressed the 10 bitter taste receptors with a coupling chimeric G protein (Gα16-gust44) in a heterologous system. For bitter taste stimuli, we chose 25 commercially available compounds, eight derived from plants, that activate human bitter taste receptors [10]. For each compound, mock-transfected cells were used as negative controls (see Methods).

**Fig 1:**
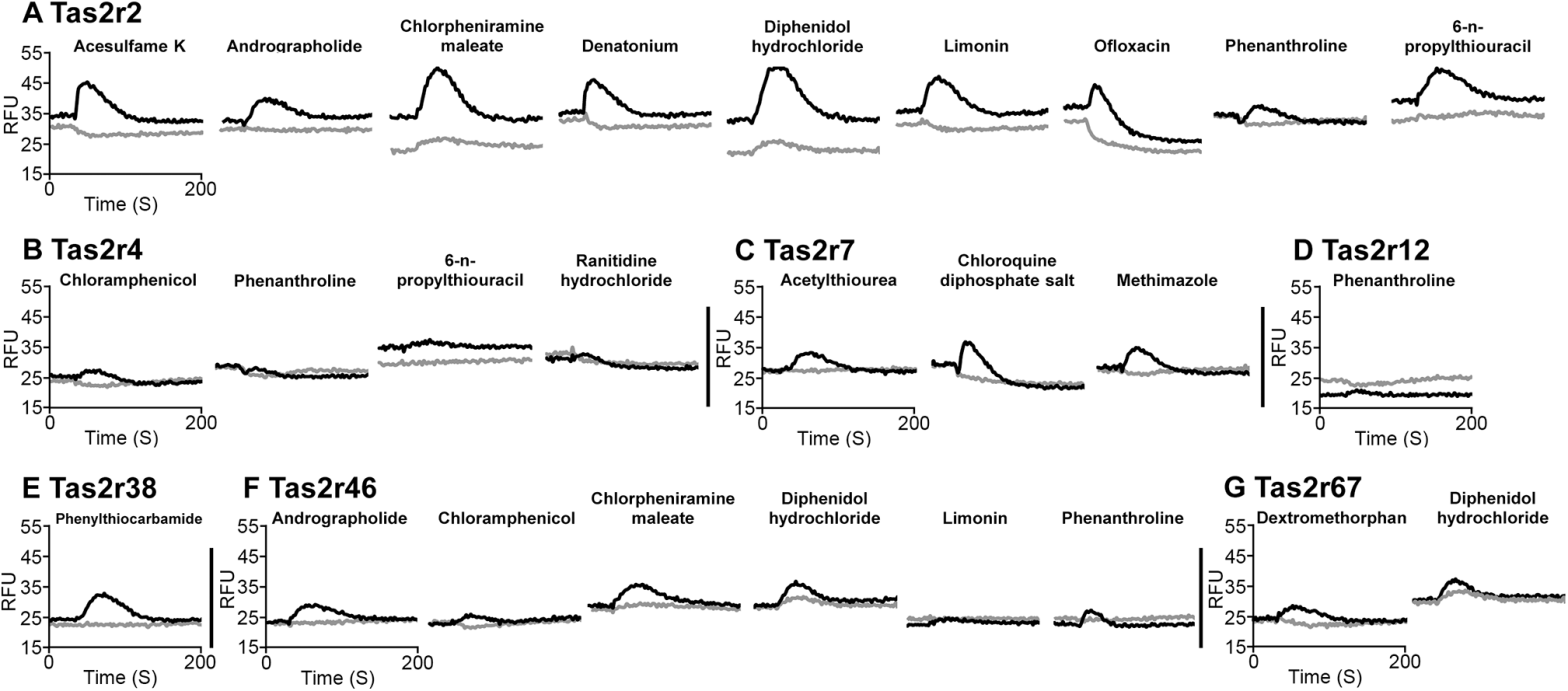
Responses of feline bitter taste receptors to bitter compounds. HEK293 cells transiently transfected with one of the feline Tas2r bitter receptors (Tas2r2, Tas2r4, Tas2r7, Tas2r12, Tas2r38, Tas2r46, or Tas2r67), with Gα16-gust44, were assayed for their responses to 25 bitter compounds. Black traces, calcium mobilization of seven feline bitter receptors to bitter compounds; grey traces, those of mock transfected cells used as control.

**Fig 2:**
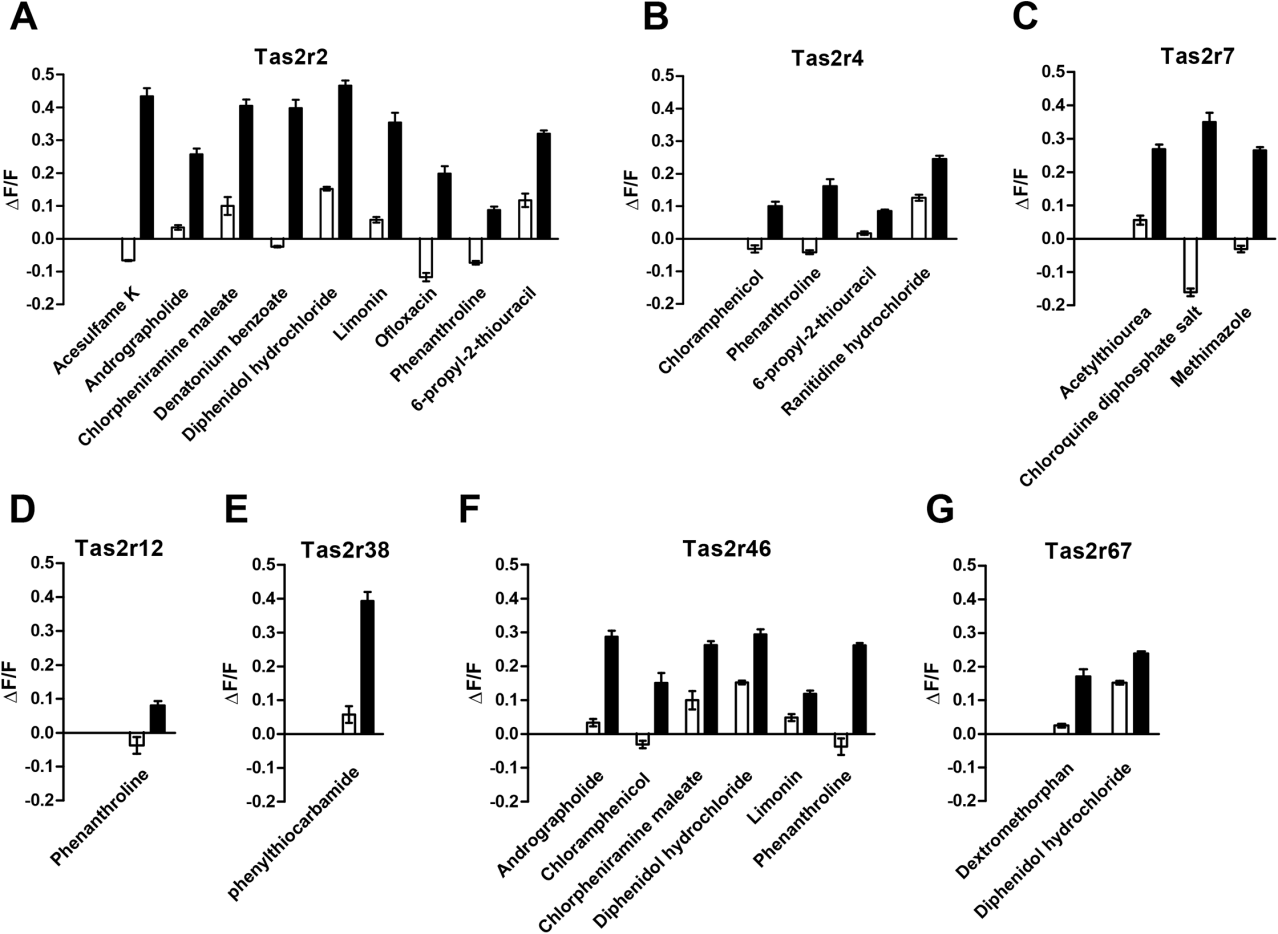
Quantitative analysis of responses of feline bitter receptors to bitter compounds. HEK293 cells were transiently transfected with a feline bitter receptor (Tas2r2, Tas2r4, Tas2r7, Tas2r12, Tas2r38, Tas2r46, or Tas2r67), with Gα16-gust44, and assayed for their responses to bitter compounds. Data are expressed as mean ± SE percent change in fluorescence (ΔF, peak fluorescence–baseline fluorescence) compared with baseline fluorescence (F) from three independent wells. Two-tailed Student’s t-tests were performed to determine when responses from Tas2r-transfected cells were significantly different from that of mock-transfect cells. Only bitter compounds that elicited significant responses above mock-transfected cell baseline are shown in the Figure.

Bitter receptors differed in the number of compounds that elicited a response. Some receptors appeared to be broadly tuned, such as Tas2r2 and Tas2r46 (**Figs 1A, 1F**, **2A and 2F**), some appeared to have an intermediate range of tuning (e.g., **Tas2r4, Figs 1B and 2B; Tas2r7; Figs 1C** and **2C; Tas2r67, Figs 1G and 2G**), and some seemed to be narrowly tuned (e.g., **Tas2r12; Figs 1D and 2D; Tas2r38; Figs 1E and 2E**). Three of the bitter receptors (Tas2r3, Tas2r9, and Tas2r42) showed no responses to any of 25 compounds tested (**S2 Table**) although this is not evidence that they are not functional; it is likely that in our small repertoire of bitter compounds tested we did not include those that activate these receptors.

**Fig 3:**
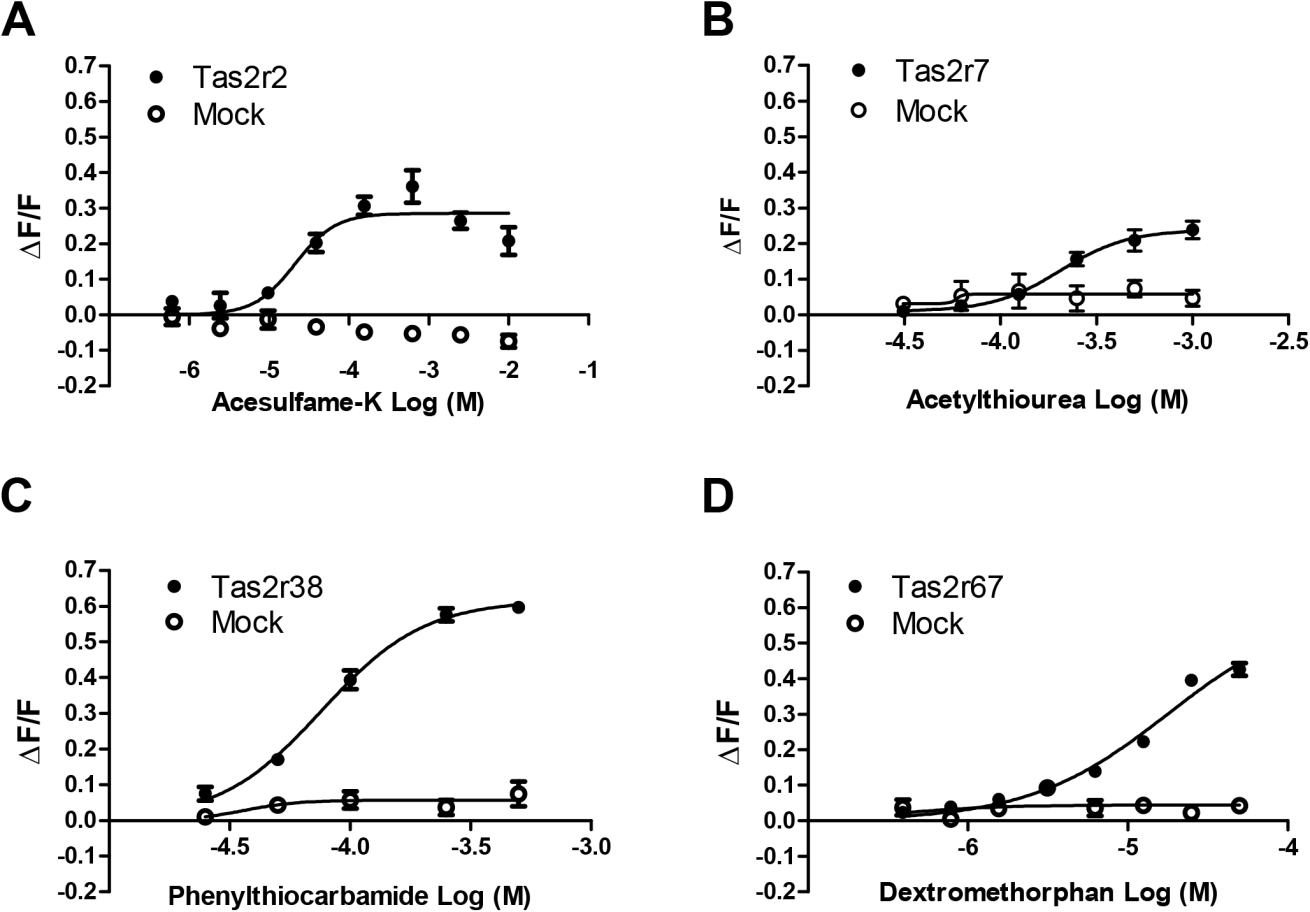
Feline Tas2rs respond to bitter compounds in a dose-dependent fashion. HEK293 cells transiently transfected with feline Tas2rs with Gα16-gust44 showed dose-dependent responses to bitter compounds. Mock-transfected cells were used as controls. The data were fit by a sigmoidal function using GraphPad Prism 5.

To confirm the validity of receptor responses, dose-dependent curves were obtained for a subset of Tas2r receptors and compounds. The Tas2r38 receptor responded to its ligand phenylthiocarbamide (PTC) in a dose-dependent manner (EC_50_ = 7.6 × 10^−5^). Dose-responsive curves were also obtained for a few additional receptors and compounds with similar results (**Fig 3**).

### A comparative perspective: Bitter receptor genes in other Carnivora

**Fig 4:**
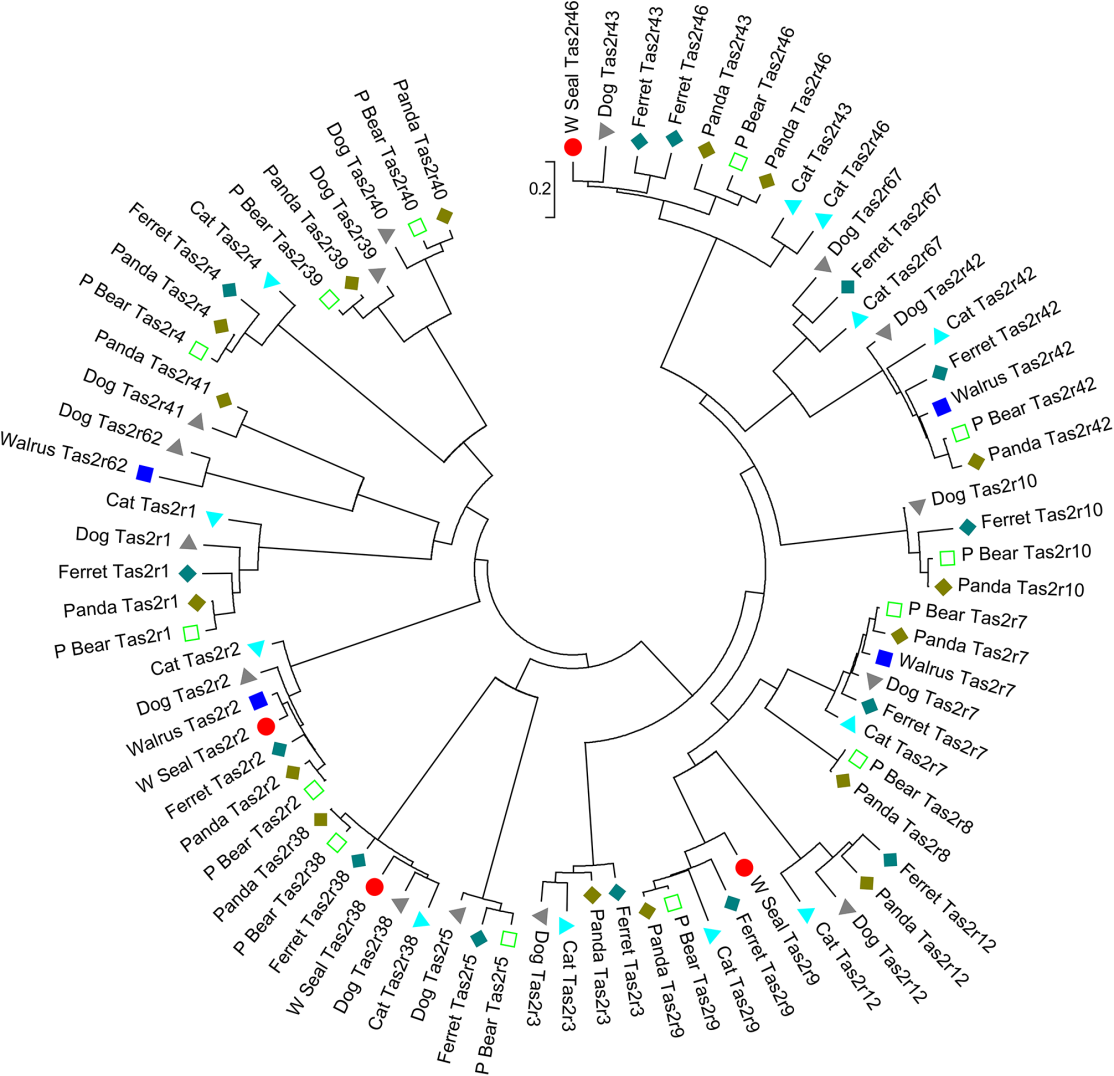
Phylogenetic tree of Tas2r repertoires of seven species in the order Carnivora. Only the predicted intact Tas2r receptors are included. Sequences were either taken from Li and Zhang or obtained from reBLASTing genomes of these seven species. The tree was constructed by using the maximum-likelihood method based on the matrix-based model of [24] implemented in Mega 6 [25]. The tree is drawn to scale, with branch lengths measured in the number of substitutions per site.

Bitter receptor gene numbers for several species were available from public sources (dog, ferret, giant panda, and polar bear; see Materials and Methods). We also extracted bitter receptor gene information from the genome of two recently sequenced species from the order Carnivora (walrus and seal) using amino acid sequences of intact bitter receptors from dogs and ferrets as queries (N = 15 sequences from dog, N = 14 sequences from ferret). We found 5 and 4 intact bitter receptor genes from walrus and seal, respectively, as well as 17 (walrus) and 11 (seal) presumably non-functional bitter receptor genes (**Tables 1 & 2**; **S1 & S2 Datasets**). This result is consistent with our previous report [3] of reduced taste receptor function in other aquatic mammals. A phylogenetic tree constructed with all predicted intact Tas2rs from seven Carnivora species (**Fig 4**, **Table 2**; see also below) shows a nearly one-to-one orthologous relationship among Tas2rs. One exclusive plant-eating Carnivora species (Giant Panda, 16 intact Tas2rs) has 1–4 more intact bitter receptors than does meat-eating species (cat, 12; polar bear, 13; ferret, 14; dog 15).

**Table 1:** Tas2r repertoires in seven species within the order Carnivora and other aquatic mammals.

Category	Tas2r bitter receptor genes				Diet type
	Intact	Partial	Pseudo-genes	Total	
Terrestrial Carnivora					
Domestic cat *(Felis catus)*	12	0	11	23	Obligate Carnivore [11, 12]
Domestic dog (*Canis lupus familiaris*)	15	0	8	23	Omnivore [12]
Ferret (*Mustela putorius furo*)	14	0	10	24	Obligate Carnivore [13]
Giant panda (*Ailuropoda melanoleuca*)	16	0	10	26	Herbivore [14]
Polar bear (*Ursus maritimus*)	13	0	11	24	Carnivore [15]
Aquatic Carnivora					
Walrus (Odobenus rosmarus)	5	0	16	21	Obligate Carnivore [16]
Seal *(Leptonychotes weddellii)*	4	2	11	17	Obligate Carnivore [17]
Other aquatic mammals					
Dolphin *(Tursiops truncatus)*	0	0	10	10	Obligate Carnivore [18]
Whale (*Balaenoptera acutorostrata)*	1	0	8	9	Obligate Carnivore [19]
Manatee *(Trichechus manatus)*	7	0	25	32	Herbivore [20]

Note: The numbers of Tas2r genes for the Dolphin, Whale and Manatee were obtained from Jiang et al. (dolphin) [3], Feng et al (whale) [21]., Zhu et al. (whale) [22], Li and Zhang (manatee) [6].

**Table 2:** Presence of Tas2r genes in seven species in the order Carnivora.

Tas2r	Cat	Dog	Ferret	Giant panda	Polar bear	Walrus	Seal
*Tas2r1*	+	+	+	+	+	ps	ps
*Tas2r2*	+	+	+	+	+	+	+
*Tas2r3*	+	+	+	+	ps	ps	ps
*Tas2r4*	+	ps	+	+	+	ps	ps
*Tas2r5*	ps	+	+	ps	+	ps	—
*Tas2r7*	+	+	+	+	+	+	partial
*Tas2r8*	ps	ps	ps	+	+	ps	—
*Tas2r9*	+	ps	+	+	+	ps	+
*Tas2r10*	ps	+	+	+	+	ps	—
*Tas2r12*	+	+	+ (122)	+	ps	ps	ps
*Tas2r38*	+	+	+	+	+	ps	+?
*Tas2r39*	ps	+	ps	+	+	ps	ps
*Tas2r40*	ps	+	ps	+	+	+?	ps
*Tas2r41*	ps	+	ps	+	ps	ps	partial
*Tas2r42*	+	+	+	+	+	+	ps
*Tas2r43*	+	+	+ (19)	+ (66*)	ps (20*)	—	—
*Tas2r46*	+	—	+ (14)	+ (31)	+	ps	+
*Tas2r62*	ps	+ (34)	ps	ps	—	+	—
*Tas2r67*	+ (18)	+	+	ps	—	ps	—

Note: Only Tas2rs with a predicted intact open reading frame in at least one species in Carnivora are listed. +, intact gene present; ps, predicted gene is pseudogenized due to either open reading frame-shifting mutations or premature stop codons;—, Tas2r ortholog is not found in the genome database from that species. Numbers in parentheses are those used in Li and Zhang [6];

*, nomenclature of predicted Tas2r in NCBI. The seal *Tas2r38* (+?) has a longer N-terminal and a predicted octahelical transmembrane domain, so it is uncertain of whether it is intact. For walrus, *Tas2r40* (+?) lacks the prototypic seven-transmembrane helices for G-protein-coupled receptors and is thus unlikely to be functional [23].

## Discussion

The goal of this study was to determine whether domestic cats, which are obligate carnivores, have multiple fully functional bitter receptors based on their activity in cell-based assays. The results confirmed that they do. Bitter receptor function in the cat exhibited similarities and some differences with that in humans. For instance, the human *TAS2R38* receptor responds to a few thiourea compounds [26], including PTC and 6-propyl-2-thiouracil (PROP). The cat Tas2r38 only responds to PTC but not PROP, at the concentrations tested. A similar observation was reported recently [9]. Likewise, human *TAS2R46* is a broadly tuned receptor that responds to a wide range of structurally diverse bitter compounds (e.g., angrographolide, chloramphenicol, chloropheniramine, denatonium benzoate, Diphenidol, Picrotoxinin, yohimbine, etc) [10], and the cat receptor was also similarly broadly tuned and responded to an overlapping set of compounds (e.g., angrographolide, chloramphenicol, chloropheniramine, diphenidol, limonin, phenanthroline). Moreover, the *Tas2r2* gene is intact in cats (and all species within Carnivora we surveyed), but it is a pseudogene in humans [27, 28].

The breadth of tuning properties of cat bitter receptors as described here are based on responses to a small set of both naturally occurring and synthetic compounds. Also, some compounds tested at higher concentration than we used may activate the receptor but were untested in this study due to non-specific responses and solubility issues. Additional work with a wider range of “bitter” compounds, especially those derived from animal products consumed by cats, will surely reveal more interesting properties of these receptors. Moreover, we note that the responses of the cat Tas2r2-transfected cells to bitter compounds appeared to have a higher baseline than that of mock-transfected cells. Although we don’t know the reason, potential constitutive activity of Tas2r2 may contribute to a higher baseline in Tas2r2-transfected cells.

To provide a comparative perspective on bitter taste receptors, we examined the number of intact bitter receptor genes in species of the order Carnivora (Tables 1 and 2). This order contains species that eat foods derived virtually exclusively from animals (obligate carnivores), eat foods derived predominantly from animals (carnivores), eat foods derived from animals and plants (omnivores), or only eat plant-based foods (herbivores). All species in this order apparently arose from a meat-eating common ancestor about 60 million years ago [29]. If bitter receptor function is strongly influenced by whether a species consumes plants, we would expect that obligate carnivores should have fewer functional bitter receptors than do herbivores perhaps through pseudogenization as happened for the sweet receptor. We found that the numbers among terrestrial Carnivora were roughly the same regardless of the amount of meat in the species’ diets although the herbivorous giant panda did have 1–4 more intact bitter receptors than meat eating carnivores (Table 1). Thus these comparative data do not provide strong support for the hypothesis that an all-meat diet would result in reduced bitter receptor number or function [6], However, due to the small number of species examined, they also do not refute this hypothesis. More comparative data are needed to critically test it.

Consistent with our previous report of major taste loss in some aquatic mammals ([3]; see also Tables 1 and 2) the two mainly water-dwelling Carnivora we examined, walrus and seal, both of which are obligate carnivores, did have a smaller number of bitter receptors (5 and 4 respectively) than the terrestrial Carnivora we examined. However, due to the small set of species available for our comparative analysis, we cannot conclude with confidence that mammals that have become sea living consistently or universally loose bitter receptor number and/or function. Nevertheless the consistency is striking. For example, in three separate lineages of mammals that have returned to aquatic life (Cetacea [dolphin, whale]; Tethytheria [manatee] and Pinnipedimorpha [walrus, seal]), all appear to have very low numbers of functional bitter receptors ([3] and Table 1). Future work with more species is warranted to further clarify the role of ecological niches (e.g., water-dwelling vs land-dwelling) in taste receptor evolution.

What factors could maintain bitter taste receptor function in terrestrial obligate carnivores? First, it could be that even obligate carnivores such as cats are actually exposed to plant material through consumption of prey viscera that contains plant material consumed by the prey. There are two arguments against this having an important role. First, plants eaten by prey may not be bitter and highly toxic since the prey species consumed them themselves. However, some species have evolved detoxification mechanisms enabling them to consume potentially toxic plants (e.g., the koala [*Phascolarctos cinereus*] feeding on the foliage of eucalyptus species, which are typically rather poisonous to most animal species) [30]. Second, the frequency that carnivores actually consume plant material in prey viscera is unclear and it has been reported, at least for wolves, that this plant-material is avoided [31].

Another possible reason for maintenance of bitter receptor number and function in cats and perhaps other carnivores is that there are also bitter compounds in many non-plant prey items in the carnivore diets (but see reference [5]). For example, domestic cats are known to feed on animal products that are also potentially bitter and toxic such as bile acids, venom and skin secretions from arthropods, reptiles and amphibians [32]. Thus our observations that bitter receptors in cats and most likely other land-dwelling Carnivora are functional could be due to selection to insure that consumption of these toxic substances is minimized.

A third reason why the number of bitter taste receptors may not be strongly influenced by the amount of dietary plant material relates to the possible non-oral functions of these receptors. Bitter receptors are found in cell types other than taste on the tongue. Neither the natural ligands nor the functions of these receptors are fully known, but we suggest they may be important in maintaining bitter receptor functionality in species that might not otherwise “need” them to avoid plant-based or animal-based toxins. For instance, respiratory-expressed bitter receptors are important for innate defense against bacterial infections [33–35].

Domestic cats have a reputation as picky eaters. Perhaps their apparently intact bitter system provides some explanation for their dietary habits. While the cell-based assays used here may not exactly recapitulate the native taste system, it allows investigators to gauge the response of the cat to bitter chemicals, including items that are poisonous, without risk to the animal. Commercial cat foods and veterinary medicines may contain bitter compounds that at least some cats find off-putting, and one way to predict their response in advance is through use of these cell-based systems.

## Materials and Methods

### Feline Tas2r receptor constructs

The coding sequences of feline Tas2r receptors were amplified from feline genome DNA using gene-specific primers (**S1 Table**). The coding sequences were inserted into an expression cassette with the first 45 amino acid residues of rat somatostatin receptor 3 as the signal peptide at the N-terminal and the herpes simplex virus glycoprotein D epitope at the C-terminal, following previously established methods [36]. All constructs were verified by direct sequencing.

### Chemicals

With the exception of acetylthiourea (Acros Organics), diphenidol hydrochloride (Reagent World), and limonin (LKT Laboratories), all chemicals were purchased from Sigma-Aldrich. For the catalog numbers and concentrations used in the assays, see **S2 Table**. Concentrations were chosen according to Meyerhof et al [10] to ensure that there were no or little unspecific responses distinguishable from that of Tas2r-transfected cells. As stated in Meyerhof et al., higher concentrations of bitter compounds could not be used because bitter compounds generated artificial calcium responses in the absence of transfected Tas2rs at high concentrations.

### Functional assays of feline bitter taste receptors

Human embryonic kidney 293 (Peakrapid) cells were obtained from ATCC and cultured in Opti-MEM supplemented with 5% fetal bovine serum. Cells were seeded in 96-well plate at a density of 50,000 per well. The next day, cells were transiently transfected with a Tas2r construct (0.1 μg/well) with a coupling chimeric G protein Gα16-gust44 (0.1 μg/well) using Lipofectamine 2000 (0.5 μl/well). After 24 h, cells were washed once with Hanks’ balanced salt solution (HBSS) and loaded with Fluo–4 for 1 h. After three washes with HBSS, cells were assayed for their responses to bitter compounds using a FlexStation III. Relative fluorescence units (excitation at 488 nm, emission at 525 nm, and cutoff at 515 nm) were read every 2 s after addition of HBSS supplemented with 2× tastants. Calcium mobilization traces were recorded.

### Data analysis

Calcium mobilization was quantified as described previously [37]. In short, changes in fluorescence (ΔF) were quantified as peak fluorescence minus the baseline level (F) and are expressed as percent ΔF relative to F, averaged from triplicate studies. Calcium mobilization traces were drawn using Excel, and bar graphs were generated using GraphPad Prism 5. Two-tailed Student’s t-tests were performed for statistical analysis.

### Genetic analysis of bitter taste receptor genes from selected species in Carnivora

The protein sequences of dog and ferret Tas2rs obtained from Li and Zhang [6] were used to query the domestic cat, seal, and walrus genomes. The retrieved sequences were analyzed for open reading frame mutations. For the intact genes, the predicted protein sequences were further examined for the presence of the seven-transmembrane helices using TMHMM methods (www.cbs.dtu.dk/services/TMHMM) [23]. The deduced amino acid sequences from cat, seal, walrus, dog, ferret, giant panda and polar bear(S1 Dataset) were aligned using Clustal X implemented in Mega 6 [25]. A phylogenetic tree among these receptors was then inferred using maximum likelihood implemented in Mega 6 [25]. All DNA sequences were provided in S2 Dataset.

## Supporting Information

S1 Dataset The predicted amino acid sequences of intact polar bear, feline, dog, ferret, giant panda, walrus, and seal Tas2r genes. (TXT)

S2 Dataset DNA sequences of Tas2rs from the polar bear, cat, dog, ferret, giant panda, walrus, and Weddell seal were presented in FASTA format. (TXT)

S1 Table Primer sequences used to amplify cat Tas2rs for cloning. (DOCX)

S2 Table Responses of cat bitter taste receptors to 25 bitter compounds (DOCX)
