# Supplementary material for: Functional Analyses of Bitter Taste Receptors in Domestic Cats (Felis catus)
Source: PLoS One. 2015 Oct 21;10(10):e0139670. doi: 10.1371/journal.pone.0139670 (PMC4619199; doi:10.1371/journal.pone.0139670)
Supplement: S1 Table — (DOCX) [file pone.0139670.s003.docx]

**S1 Table**. Primer sequences used to amplify cat Tas2rs for cloning.

| **Tas2r** | **Primer** | **Sequence (5’-3’)** |
| --- | --- | --- |
| **Tas2r1** | Forward | ACGGCAAGCGCTATGCTAGACTTTTACCTCATTATC |
|  | Reverse | ACGGCACCGCGGCCTGGCAGCACTGCCCATGGAGGAG |
|  |  |  |
| **Tas2r2** | Forward | ACGGCAAGCGCTATGGCCTCCTCTTTGTCAGCGATT |
|  | Reverse | ACGGCACCGCGGCTTGATTCTTTTTGAGGCAGAGAAG |
|  |  |  |
| **Tas2r3** | Forward | CCTGGCAGGACTGGCTGTCAGTGGCATGTCAGGGCTCCACAAGTGGGTG |
|  | Reverse | ACGGCAGCGGCCGCTCAATCCTCGGGGTCTTCCGGGGCGAGTTCTGGCTG  CAGGGAAACAGGTCCTTTGGATCC |
|  |  |  |
| **Tas2r4** | Forward | ACGGCAAGCGCTATGCATCAGATACTCTTCTTATCT |
|  | Reverse | ACGGCACCGCGGCTTTACTACTGAAATTCCACCATAT |
|  |  |  |
| **Tas2r7** | Forward | ACGGCAAGCGCTATGCTGGATAAAGTGGAGAGCACC |
|  | Reverse | ACGGCACCGCGGCGTGATTTCTTCTTTTTAGGATACA |
|  |  |  |
| **Tas2r9** | Forward | CCTGGCAGGACTGGCTGTCAGTGGCATGCCAAGTGCAGTGGAGGTAATA |
|  | Reverse | ACGGCAGCGGCCGCTCAATCCTCGGGGTCTTCCGGGGCGAGTTCTGGCTG  AGCAAATGAATGTAACCCCCGGGG |
|  |  |  |
| **Tas2r12** | Forward | ACGGCAAGCGCTATGGCAAGCGTATTGAAGAATGTA |
|  | Reverse | ACGGCACCGCGGCTGGGCGTGTGGGTTTCCTTAGATG |
|  |  |  |
| **Tas2r38** | Forward | ACGGCAAGCGCTATGTTGGCTCTGACTCCTGTCATA |
|  | Reverse | ACGGCACCGCGGCACATAGGCCTGGAGTCCTGGCATC |
|  |  |  |
| **Tas2r42** | Forward | ACGGCAAGCGCTATGTTAGCCGGACTGGATAAAATC |
|  | Reverse | ACGGCACCGCGGCGAAAGACCCTGGAACGTCTATCTG |
|  |  |  |
| **Tas2r43** | Forward | ACGGCAAGCGCTATGGTAACCGCGCTACCG |
|  | Reverse | ACGGCACCGCGGCGCACTTCAGCTGCCACAA |
|  |  |  |
| **Tas2r46** | Forward | ACGGCAAGCGCTATGGTAAGCGCGCTACCAAGCATT |
|  | Reverse | ACGGCACCGCGGCTGGAGTTGAGTGTTTCTGTTCTTT |
|  |  |  |
| **Tas2r67** | Forward | ACGGCAAGCGCTATGCCATCTGGAATCGAAAATACT |
|  | Reverse | ACGGCACCGCGGCTGAAGCTAAAGGTTTCACCATTTT |

Note: Underlined nucleotides are introduced for cloning purposes
