# Supplementary material for: Functional Analyses of Bitter Taste Receptors in Domestic Cats (Felis catus)
Source: PLoS One. 2015 Oct 21;10(10):e0139670. doi: 10.1371/journal.pone.0139670 (PMC4619199; doi:10.1371/journal.pone.0139670)
Supplement: S2 Table — (DOCX) [file pone.0139670.s004.docx]

**S2 Table**. Responses of cat bitter taste receptors to 25 bitter compounds

| **Compound** | **Catalog#** | **[mM]** | **2** | **3** | **4** | **7** | **9** | **12** | **38** | **42** | **46** | **67** |
| --- | --- | --- | --- | --- | --- | --- | --- | --- | --- | --- | --- | --- |
| acesulfame K | 04054 | 10 | + | - | - | - | - | - | - | - | - | - |
| acetylthiourea | 591-08-2 | 0.3 | - | - | - | + | - | - | - | - | - | - |
| amygdalin  (plant) | A6005 | 30 | - | - | - | - | - | - | - | - | - | - |
| andrographolide (plant) | 365645 | 0.3 | + | - | - | - | - | - | - | - | + | - |
| arbutin  (plant) | A4256 | 30 | - | - | - | - | - | - | - | - | - | - |
| chloramphenicol | C0378 | 1 | - | - | + | - | - | - | - | - | + | - |
| chloroquine diphosphate salt | C6628 | 10 | - | - | - | + | - | - | - | - | - | - |
| chlorpheniramine maleate | C3025 | 0.1 | + | - | - | - | - | - | - | - | + | - |
| denatonium benzoate | D5765 | 5 | + | - | - | - | - | - | - | - | - | - |
| dextromethorphan | 1180503 | 0.01 | - | - | - | - | - | - | - | - | - | + |
| diphenidol hydrochloride | 5F4-06 | 0.1 | + | - | - | - | - | - | + | - | + | + |
| limonin  (plant) | L3550 | 0.3 | + | - | - | - | - | - | - | - | + | - |
| methimazole | M8506 | 10 | - | - | - | + | - | - | - | - | - | - |
| ofloxacin | O8757 | 4 | + | - | - | - | - | - | - | - | - | - |
| papaverine  (plant) | P3510 | 0.01 | - | - | - | - | - | - | - | - | - | - |
| phenanthroline | 131377 | 1 | + | - | + | - | - | + | - | - | + | - |
| picrotoxinin  (plant) | P8390 | 1 | - | - | - | - | - | - | - | - | - | - |
| 6-n-propylthiouracil | P3755 | 1 | + | - | + | - | - | - | - | - | - | - |
| phenylthiocarbamide | P7629 | 0.1 | - | - | - | - | - | - | + | - | - | - |
| quinine | Q1125 | 0.01 | - | - | - | - | - | - | - | - | - | - |
| ranitidine hydrochloride | R101 | 10 | - | - | + | - | - | - | - | - | - | - |
| saccharin | 240931 | 10 | - | - | - | - | - | - | - | - | - | - |
| salicin  (plant) | S0625 | 3 | - | - | - | - | - | - | - | - | - | - |
| sodium thiocyanate | S7757 | 3 | - | - | - | - | - | - | - | - | - | - |
| yohimbine  (plant) | Y3125 | 0.3 | - | - | - | - | - | - | - | - | - | - |

Note: Numbers 2 -67 refer to Tas2r genes. + indicates a positive response.- indicates a negative response at the concentration listed. Compounds derived from plants are specifically indicated, the remainders are synthetic.
